# Pulsed electromagnetic stimulation in nonunion of tibial diaphyseal fractures

**DOI:** 10.4103/0019-5413.50850

**Published:** 2009

**Authors:** Anil Kumar Gupta, Kailash Prasad Srivastava, Sachin Avasthi

**Affiliations:** Department of Orthopaedic Surgery, GSVM Medical College, Kanpur, India; 1 Department of Orthopaedic Surgery, SN Medical College, Agra, India

**Keywords:** Nonunion, pulsed electromagnetic stimulation, tibial diaphyseal fractures

## Abstract

**Background:**

Nonunion of long bones is a difficult clinical problem and challenges the clinical acumen of surgeons. Multiple surgical or nonsurgical modalities have been used to treat nonunions. Noninvasive pulsed electromagnetic stimulation is an entity known to affect the piezoelectric phenomenon of bone forming cells. We conducted a study on 45 long-bone fractures of tibia treated by pulsed electromagnetic stimulation, which are analyzed and reported.

**Materials and Methods:**

A total of 45 tibial fractures with established atrophic nonunion were enrolled between 1981 and 1988. All the patients had abnormal mobility and no or minimal gap at fracture site with no evidence of callus formation across the fracture site. The patients' age ranged between 24 and 68 years; 40 were men and 5 were women. All patients having evidence of infection, implant *in situ*, and gap nonunions were excluded from study. Pulsed electromagnetic stimulation was given using above-knee plaster of Paris cast (0.008 Weber/m2 magnetic field was created for 12 h/day). The average duration for pulsed electromagnetic stimulation (PEMS) therapy was 8.35 weeks, with the range being 6–12 weeks. The cases were evaluated at 6 weeks and subsequently every 6-weekly interval for clinical and radiological union. The withdrawal of therapy was decided as per clinicoradiological evidence of union.

**Results:**

All but three patients showed evidence of union. About 35% (n = 16) cases showed union in 10 weeks, and 85%(n = 38) cases showed union in 4 months. The average duration of therapy using PEMS was 8.35±0.48 weeks, and the average duration of immobilization was 3.02 ± 0.22 months. Three cases that did not show evidence of union were poorly compliant for the apparatus of PEMS.

**Conclusion:**

PEMS is a useful noninvasive modality of treatment for difficult nonunion of long bones.

## INTRODUCTION

Nonunion of long bones is a challenge for orthopedic surgeons.^1^–^3^ A diagnosis of nonunion is unjustified until there is evidence, either clinical or radiological, that healing has ceased and that union is highly improbable. The exact causes of nonunion are unknown, but various factors contribute to this unwanted complication.^4^ Many surgical and nonsurgical modalities have been proposed to reduce the chances of nonunion or to treat the established nonunion. Discovery of electrical phenomenon in skeletal system and its cellular environment has led to the establishment of techniques of electrical stimulation in the promotion of bone healing.^1^–^3^ This was followed by a series of studies to establish the role of electrical stimulation in fracture healing^5^–^11^ Noninvasive methodology to generate electrical field around the bone defect has gained popularity, as a number of problems were envisioned in invasive method, which includes the insertion of multiple electrodes and iatrogenic infection. Change of electrical environment around fibroblast has led to the application of the noninvasive method for changing the electrical environment around the cell.^12^ This led to a series of experiments to establish the method as a modality for therapeutic use.^13^–^16^ The therapeutic potential of this method was proved by conducting animal studies.^17^,^18^ Also, clinical trials were conducted to establish the outcome of the modality.^11^,^19^,^20^ Considering the above facts, we hypothesized that pulsed electromagnetic stimulation (PEMS) is a safe and effective modality for conservatively treating nonunion of long bones with the null hypothesis being that it does not have any effect. We conducted a study of 45 tibial nonunion cases treated by PEMS, and the results were analyzed to determine its efficacy.

## MATERIALS AND METHODS

This study was started in 1981 and continued till 1988 simultaneously at two large tertiary-level teaching hospitals. A total of 45 cases of nonunion tibia were enrolled and subsequently treated by electrical stimulation. Established nonunion of tibia (defined as >9 months duration since injury with no evidence of radiological progression of union in the past 3 months) were included in the study. Criteria for inclusion were no or minimal gap at fracture site and insufficient callus formation. However, we excluded the cases having evidence of osteomyelitis or where implant was placed in situ due to primary management. The nonunion was mobile in all the cases, with the absence of bridging callus across the fracture site as seen on radiography.

**Figure 1:**
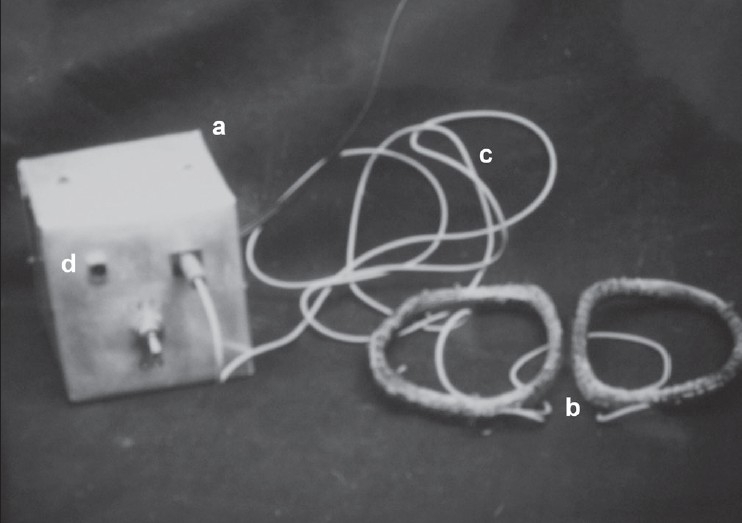
Photograph showing the pulsing electro magnetic stimulator: (a) pulse generator, (b) pair of coils (c) connecting wire to main equipment box (d) indicator

All the patients selected were subjected to routine clinical and laboratory examination. The possible causes of poor healing were established by excluding any systemic defects such as diabetes mellitus, malnutrition, alcoholism, and tobacco smoking. The cases of nonunion having satisfactory axial and rotatory alignment of fracture were immobilized using a well fitting long leg plaster. Cases not showing satisfactory axial or rotatory alignment were excluded. The acceptable reduction was defined as >50% contact across bone ends in AP and lateral views. No rotation or angulation was accepted. The fracture site was marked over the skin of the opposite limb before applying plaster. The diameter of cast was measured to determine the distance between two coils. A pair of oval-shaped coils was put across the fracture site centering over the fracture at 180° to each other [Figure 1]. Radiological confirmation of position of coil was done. The pair of coils was then connected to the main equipment box, which operated at 220 V AC. It consisted of a step down transformer, pulse generator, and a power amplifier [Figure 1]. The transformer converted the 220 V AC current to 30 V DC current and pulse generator converts it into pulsing current. It delivers 0.3-ms quasirectengular pulse with a repetition rate of 75 Hz. Amplifier amplifies it and delivers to the pair of coils. The specific intercoil distance with parallel coil creates a Helmholtz aiding effect, i.e., production of uniform pulsing electromagnetic field at all points within the area of bone to be treated.^21^

**Figure 2:**
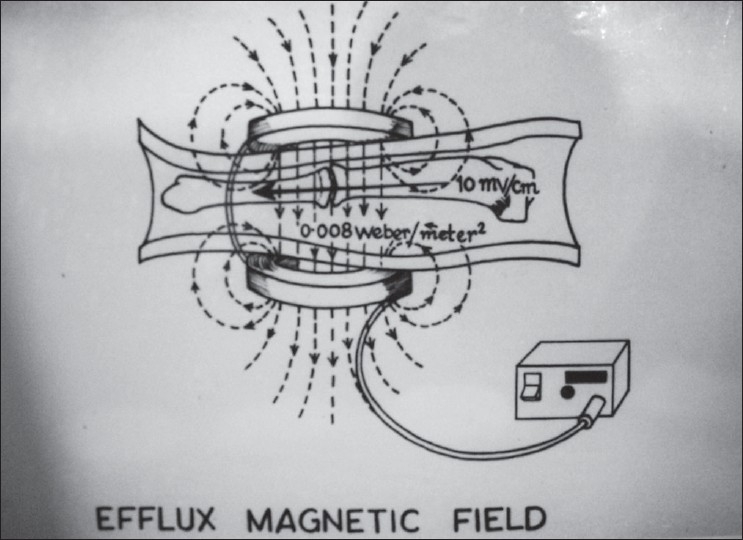
Diagramatic representation of the pair of coils, lines of electromagnetic field, fracture site, pulse generator and connector. As current flows in coils, pulsing electromagnetic field of 0.008 webers/meter square intensity establishes between the pair of coils producing the voltage drop 10 mv/cm at right angle to field in longitudinal axis of tibia

**Figure 3:**
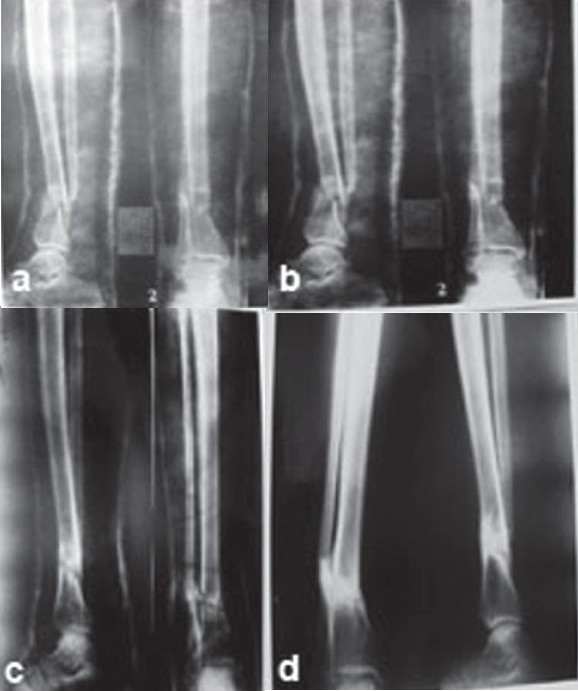
Sequential x-rays of leg bones (antero posterior and lateral views) of a 10 months old, initially compound fracture of distal one fourth of the bone of leg in a 34 year old male, at pretreatment stage (a); after 6 weeks of PEMS showing stage one healing (b); after 12 weeks PEMS showing stage and PEMS was discounted (c); 24 weeks after the star of PEMS showing established lamellar pattern and bone healing (d).

Overall a 0.008 Weber/m^2^ magnetic field was created across the fracture site, and PEMS was given for 12 h/day at a stretch [Figure 2]. Absolute non–weight-bearing was maintained for 10–12 weeks. Patients were immobilized in patellar tendon bearing Plaster of Paris cast with walking iron at 3 months, and the cast support was removed only after complete union. Clinicoradiological assessment of fracture healing was done at 6-weekly intervals. Patients were analyzed for the absence of tenderness and presence of transmitted movements. Radiologically, in stage I healing, calcified callus was considered to be the sign of union, which was evident by fuzziness in fracture gap with patchy sclerosis. Stage II healing was indicated by the appearance of consolidated bone stress lines bridging the fracture gap. Stage III healing was indicated by medullarization of fracture gap. Remodeling was considered as stage IV healing [Figure 3a–d]. The results were evaluated at 6 weeks and 6-weekly intervals thereafter. Patients were followed up till 9 months, and absence of complete union as described above was taken as a failure. If, during the first 12 weeks of treatment, stage I healing was not achieved radiologically, then this modality of treatment was considered as failure, and treatment was abandoned.

## RESULTS

Forty-five cases of nonunion tibia were enrolled and underwent treatment. The youngest patient was of 24 years and oldest was of 68 years, with average age being 42.8 years. 90% (n = 40) of cases were males and the rest were females (n = 5). In our series, 28 cases had fracture in the middle third of tibia, and 17 cases had fracture in the lower third of tibia. Out of them, 20% (n = 9) cases were initially compound (grades I and II), and the rest were simple cases of fracture of leg bones. Improper immobilization and reduction were the main factors leading to nonunion in 85% of cases (n = 38). Excessive comminution (more than 4 bony fragments apart from two major fragments) was noted in 20% (n = 9) of cases. Nine cases had systemic factors predisposing for nonunion such as diabetes mellitus, malnutrition, alcoholism, and tobacco smoking. About 70% (n = 31) cases suffered trauma as a result of road traffic accident, and the rest were due to domestic trauma.

The average duration since trauma until we started electrical stimulation was of 9.08 ± 0.22 months (9–11 months). About 31% (n = 14) of the cases showed only abnormal mobility as presenting feature, and 69% (n = 31) showed abnormal mobility and pain on bending stress. PEMS was applied in all the cases as detailed above. The average duration of therapy using PEMS was 8.35 ± 0.48 weeks.

First radiological evidence of the start of union appeared at 6 weeks in all the cases except one case, in which it appeared around 12 weeks. Complete union was achieved in 16 (35%) cases within 10 weeks and in 38 (85%) cases by 4 months. Average duration of immobilization was 3.02 ± 0.22 months, and time of union was 10–42 weeks. Out of the rest 7 (15 %) cases, union was achieved in 4 cases in 6 months, but their electrical stimulation was stopped after 3 months. There was evidence of callus formation, but it was not fully calcified. Three cases did not show evidence of union. Complications noted include increase in joint stiffness in all the patients compared with pretreatment status and persistent nonunion in three cases. Poor compliance to the electromagnetic device was thought to be the most common reason for failure of therapy.

## DISCUSSION

Nonunion that has not progressed to pseudoarthrosis can be effectively treated by the generation of electromagnetic field around fracture ends. This statement is based on the established facts regarding the modification of cellular behavior in and around the bone using noninvasive electromagnetic stimulation.^6^,^21^ The evidence of osteoblast stimulation was given in 1965 when it was proved that bone components have electromagnetic environment, which if modulated can lead to improved osteogenesis.^22^ By the production of time-varying magnetic field, it is possible to induce current in tissues that is put within it. PEMS promotes organization and consolidation in delayed union. Osteoblast realignment and stimulation for callus formation are the probable answers. Diniz *et al*. studied the role of pulsed electromagnetic field (PEMF) stimulatory effect on bone tissue formation. Their question was whether it was associated with the increase in the number of cells and/or with the enhancement of the cellular differentiation. The cellular proliferation (DNA content), differentiation (alkaline phosphatase activity), and bone tissue-like formation (area of mineralized matrix) were determined at different time points. PEMF treatment of osteoblasts in the active proliferation stage accelerated cellular proliferation, enhanced cellular differentiation, and increased bone tissue-like formation. PEMF treatment of osteoblasts in the differentiation stage enhanced cellular differentiation and increased bone tissue-like formation.^23^ Preclinical studies have demonstrated that PEMS has role in stimulating protein synthesis, which have effect in gene regulation.^23^ Upregulation of mRNA levels and protein synthesis for growth factors has also been observed with the application of PEMS.^24^ This enhances cellular repair and synthesis of extracellular proteins, which have role in differentiation and growth. Amplification of role of PEMS is probably due to transmembrane receptors such as IL-2, IGF-2, calcitonin, PTHs, etc.^25^

Considering the above fact, a number of series have been developed and people have proved the evidence of bone union by PEMS. Dehauss *et al*. (1980),^26^ Bassett *et al*. (1981),^21^ Sharrad *et al*. (1982),^27^ Satter *et al*. (1999),^28^ Abeed *et al*. (1998),^29^ and Zamora Navas *et al*. (1995)^30^ have given their series and the comparison of present series has been done [Table 1].

**Table 1:** Comparison of various series

Series	Figure	Avg. age (years)	Avg. pretreatment duration (months)	Duration of treatment h/day
Bassett *et al.* (1982)	125	36	28	10
Sharrad *et al.* (1982)	53	37	28	12.16
Dehauss *et al.* (1980)	17	34	22.2	22
Zamora Navas *et al.*	22	32	26	-
Abeed RI *et al.* (1998)	16	-	30 weeks	-
Satter *et al.* (1999)	19	30	41.3 weeks	-
Our series	45	42.8	9.08 ± 0.22	12

Comparison of above data shows that PEMS gives excellent outcome in all parts of world. Recent series of Simons *et al*.(2003)^31^ and Punt *et al*.(2008)^32^ has also shown that it is an effective way of treatment of nonunion tibia. However, daily duration of therapy varies in different studies. We found 12 hrs / day schedule to be effective and compliant for patients as well. In our series, >90% success was achieved which is at par with other series in the world. We found that infection has no detrimental effect on fracture healing but gap between fracture ends and distance between coils are important factors. In our protocol the distance between coils has worked by producing coil effects across fracture ends. They have worked by generation of electromagnetic field leading to stimulation of osteosynthesis. The idea that PEMS can be used as stimulant of bone healing in fresh fractures cannot be denied considering the physiological basis and generation of evidence of promoting bone healing in nonunion cases. Their role in fresh fractures needs to be studied further.In our opinion the best nonunion to be treated by this method is one without any other complication such as infection, etc. Compound fractures in need of dressing with gap or loss of bony fragment will be difficult to be managed by this modality.

## CONCLUSION

PEMS is a safe and effective modality of treatment in cases of nonunion. Judicious application and vigilant follow-up leads to excellent bone union across bone ends.
